# Upregulation of NOD1 and NOD2 contribute to cancer progression through the positive regulation of tumorigenicity and metastasis in human squamous cervical cancer

**DOI:** 10.1186/s12916-022-02248-w

**Published:** 2022-02-08

**Authors:** Yuanyuan Zhang, Ning Li, Guangwen Yuan, Hongwen Yao, Die Zhang, Nan Li, Gongyi Zhang, Yangchun Sun, Wenpeng Wang, Jia Zeng, Ningzhi Xu, Mei Liu, Lingying Wu

**Affiliations:** 1 Department of Gynecologic Oncology, National Cancer Center/ National Clinical Research Center for Cancer/ Cancer Hospital, Chinese Academy of Medical Sciences & Peking Union Medical College, Panjiayuan, Chaoyang District, 100021 Beijing, People’s Republic of China; 2 Laboratory of Cell and Molecular Biology & State Key Laboratory of Molecular Oncology, National Cancer Center/ National Clinical Research Center for Cancer/ Cancer Hospital, Chinese Academy of Medical Sciences & Peking Union Medical College, Panjiayuan, Chaoyang District, 100021 Beijing, People’s Republic of China

**Keywords:** Cervical squamous cell carcinoma, NOD1/2, Tumorigenicity, Metastasis, IL-8

## Abstract

**Background:**

Metastatic cervical squamous cell carcinoma (CSCC) has poor prognosis and is recalcitrant to the current treatment strategies, which warrants the necessity to identify novel prognostic markers and therapeutic targets. Given that CSCC is a virus-induced malignancy, we hypothesized that the pattern recognition receptors (PRRs) involved in the innate immune response likely play a critical role in tumor development.

**Methods:**

A bioinformatics analysis, qPCR, IHC, immunofluorescence, and WB were performed to determine the expression of NOD1/NOD2. The biological characteristics of overexpression NOD1 or NOD2 CSCC cells were compared to parental cells: proliferation, migration/invasion and cytokines secretion were examined in vitro through CCK8/colony formation/cell cycle profiling/cell counting, wound healing/transwell, and ELISA assays, respectively. The proliferative and metastatic capacity of overexpression NOD1 or NOD2 CSCC cells were also evaluated in vivo. FCM, mRNA and protein arrays, ELISA, and WB were used to identify the mechanisms involved, while novel pharmacological treatment were evaluated in vitro and in vivo. Quantitative variables between two groups were compared by Student’s *t* test (normal distribution) or Mann-Whitney *U* test (non-normal distribution), and one-way or two-way ANOVA was used for comparing multiple groups. Pearson *χ*^2^ test or Fisher’s exact test was used to compare qualitative variables. Survival curves were plotted by the Kaplan-Meier method and compared by the log-rank test. *P* values of < 0.05 were considered statistically significant.

**Results:**

NOD1 was highly expressed in CSCC with lymph-vascular space invasion (LVSI, *P* < 0.01) and lymph node metastasis (LM, *P* < 0.01) and related to worse overall survival (OS, *P* = 0.016). In vitro and in vivo functional assays revealed that the upregulation of NOD1 or NOD2 in CSCC cells promoted proliferation, invasion, and migration. Mechanistically, NOD1 and NOD2 exerted their oncogenic effects by activating NF-κb and ERK signaling pathways and enhancing IL-8 secretion. Inhibition of the IL-8 receptor partially abrogated the effects of NOD1/2 on CSCC cells.

**Conclusions:**

NOD1/2-NF-κb/ERK and IL-8 axis may be involved in the progression of CSCC; the NOD1 significantly enhanced the progression of proliferation and metastasis, which leads to a poor prognosis. Anti-IL-8 was identified as a potential therapeutic target for patients with NOD1^high^ tumor.

**Supplementary Information:**

The online version contains supplementary material available at 10.1186/s12916-022-02248-w.

## Background

Cervix carcinoma is the most common malignancy of the reproductive tract in females [1, 2], and developing countries account for 85–90% of the newly diagnosed cases and deaths every year [1, 3]. The WHO Cervical Cancer Elimination Modelling Consortium (CCEMC) has been established to eliminate cervical cancer in the low-income and lower middle-income countries through regular screening and human papilloma virus (HPV) vaccination [4–6]. The burden of cervical cancer is especially high in China, and over 106,000 new cases and 48,000 deaths have been reported in 2018 [2, 7, 8]. HPV infection-induced cervical squamous cell carcinoma (CSCC) is the predominant pathological subtype of cervical cancer [3, 9]. Since vaccination is estimated to achieve only a 0.1–0.5% reduction in mortality rates until 2030 [5, 10], there is an urgent need for novel treatment strategies. Therefore, it is necessary to elucidate the molecular mechanisms involved in the progression of CSCC in order to identify potential therapeutic targets. Given the vital role of HPV in cervical carcinogenesis, the correlation between immunological factors and cancer progression needs to be investigated.

Pattern recognition receptors (PRRs) are host sensors that detect pathogen-specific molecules and act as the first line of defense against infections. The toll-like receptors (TLRs) and nucleotide-binding oligomerization domain receptors (NODs) are the two major PRRs expressed on/in the cells that recognize invading pathogens and mediate the inflammatory response [11–13]. NOD1 and NOD2 recognize pathogens that express meso-diaminopimelic acid (meso-DAP) and muramyl dipeptide (MDP) respectively [11, 14, 15]. Recent studies have implicated PRRs in the carcinogenesis of multiple tissues. TLR4 and TLR2 enhance metastasis of colon cancers [16–18], whereas NOD1 promotes several gastrointestinal malignancies [19] such as colon cancer [20], as well as head and neck and oral squamous cell carcinoma [21, 22]. In addition, higher baseline levels of TLR2 and TLR7 are associated with prior clearance of HPV in women with cervical intra-epithelial (CIN) 2 lesions [23]. TLR5 is overexpressed in high-grade cervical dysplasia and invasive cancers but is commonly absent in the normal cervix [24]. TLR2 and TLR9 show significant variation in their expression levels in CSCC [25]. Furthermore, the TLR9 agonist CpG oligodeoxynucleotide (CpG ODN) can effectively treat solid tumors in combination with rlipo-E7m [26]. In contrast, the role of NODs in cervical cancer progression is unclear. A recent study showed that downregulation of NOD1 promoted CIN progression to cervical cancer [27]. In this study, we examined the expression of the NOD family of proteins in CSCC tissues and cell lines to gain further insights into their role in advanced cervical malignancies.

## Results

### NOD1 is overexpressed in CSCC tissues and associated with poor prognosis

**Fig. 1:**
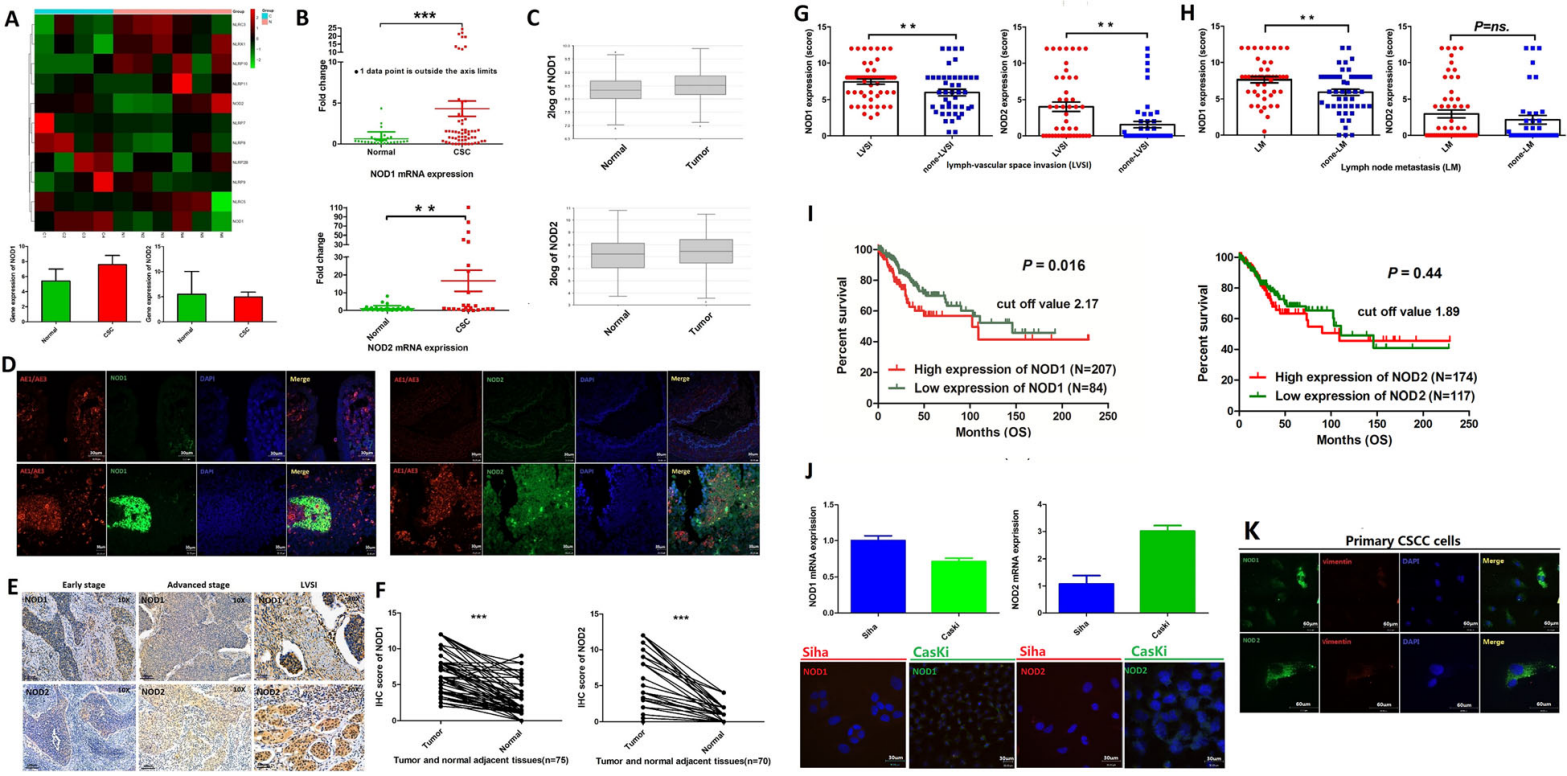
NOD1 and NOD2 are upregulated in human CSCC tissues and associated with poor survival. **A** Partial heat map showing differentially expressed NLR genes including NOD1 and NOD2 in the CSCC (*n* = 4) and normal cervix (*n* = 6) tissues. **B** NOD1 and NOD2 mRNA copy numbers in unpaired CSCC tissues (NOD1, *n* = 59; NOD2, *n* = 24) and normal cervix (NOD1, *n* = 33; NOD2, *n* = 31). **C** NOD1 and NOD2 mRNA expression in the CSCC (NOD1, *n* = 75; NOD2 *n* = 75) and normal cervix samples (non-tumoral adjacent tissue, NOD1, *n* = 188; NOD2 *n* = 188) extracted from TCGA database. **D** Representative immunofluorescence images showing co-staining of AE1/AE3 and NOD1/NOD2 in paired CSCC tumors and normal cervix tissues (data were from two independent experiments with eight samples). **E** Representative IHC images showing in situ expression of NOD1and NOD2 in paired human CSCC tissues of different pathological stages (early and late stages and LVSI) and adjacent non-tumor tissues (scale bar = 100 μm and magnification—× 10 or × 20). **F–H** IHC scores of NOD1 and NOD2 in **F** paired tumor and adjacent non-tumor tissues (NOD1, *n* = 75; NOD2, *n* = 70), **G** tumors with and without LVSI (NOD1: LVSI = 58, non-LVSI = 45; NOD2: LVSI = 48, non-LVSI = 49), and **H** tumors with and without LM (NOD1: LM = 48, non-LM = 48; NOD2: LM = 55, non-LM = 39). **I** Kaplan-Meier curves showing overall survival of CSCC patients demarcated on the basis of in situ NOD1 expression (http://www.proteinatlas.org). **J** NOD1 and NOD2 mRNA levels and representative immunofluorescence images showing respective protein levels in Siha and CasKi cell lines. **K** Immunofluorescence images showing respective NOD1 and NOD2 protein levels in primary CSC cells. For cell lines, the experiments were performed in two wells with three replicates; for primary cells, the experiments were performed by two independent experiments with four samples; the picture is a representative one. *P < 0.05, ** *P* < 0.01, *** *P* < 0.001

The CSCC tissues were confirmed by histopathological examination and immunostaining for specific markers (Additional file 1: Fig.S1A). Bioinformatics analysis identified 5140 upregulated genes in the CSCC samples, including NOD1 and NOD2 (Additional file 1: Fig.S1B). Preliminary RNA-Seq analysis confirmed that NOD1 and NOD2 were upregulated in the CSCC relative to normal cervix tissues (Fig. 1A), which was further confirmed by RT-PCR (Fig. 1B) and analysis of TCGA data (Fig. 1C). Consistent with this, the NOD1 and NOD2 protein levels were significantly higher in the CSCC tissues compared to the paired adjacent normal cervix tissues (Fig. 1D–F). Furthermore, both NOD1 and NOD2 were overexpressed in embolic tumor cells resulting from lymph-vascular space invasion (LVSI) compared to the primary tumors without LVSI (*P* < 0.05, Fig. 1G), whereas significantly higher expression of NOD1 was detected in the CSCC tissues of patients with lymph node metastasis (LM) relative to the non-LM samples (*P* < 0.05, Fig. 1H). Although the tumor stage was not associated with NOD1/NOD2 expression, both were overexpressed in tumors with poorer differentiation (Additional file 1: Fig.S1C and S1D). In clinic practice, LVSI and LM were risk factors for un-promising survival trend. In agreement with the above findings, TCGA data showed that overexpression of NOD1 predicted a worse prognosis in CSCC patients (Fig. 1I, left panel), whereas NOD2 expression level did not show significantly correlation with the overall survival (Fig. 1I, right panel). Interestingly, while the NOD1 and NOD2 mRNA levels showed a positive correlation (Additional file 1: Fig.S1E); the TCGA data showed that both overexpression of NOD1 and NOD2 predicted a worse prognosis trend in CSCC patients but not reached significance. These results maybe according to the small cohort group (Additional file 1: Fig.S1F). Consistent with the patient samples, NOD1/NOD2 mRNA and protein levels were intrinsically expressed in the CSCC cell lines (Fig. 1J) and primary CSCC cells (Fig. 1K). In conclusion, NOD1 may play an important role in the progression of CSCC patients.

### NOD1 and NOD2 promoted CSCC cell proliferation and enhanced metastatic potential

**Fig. 2:**
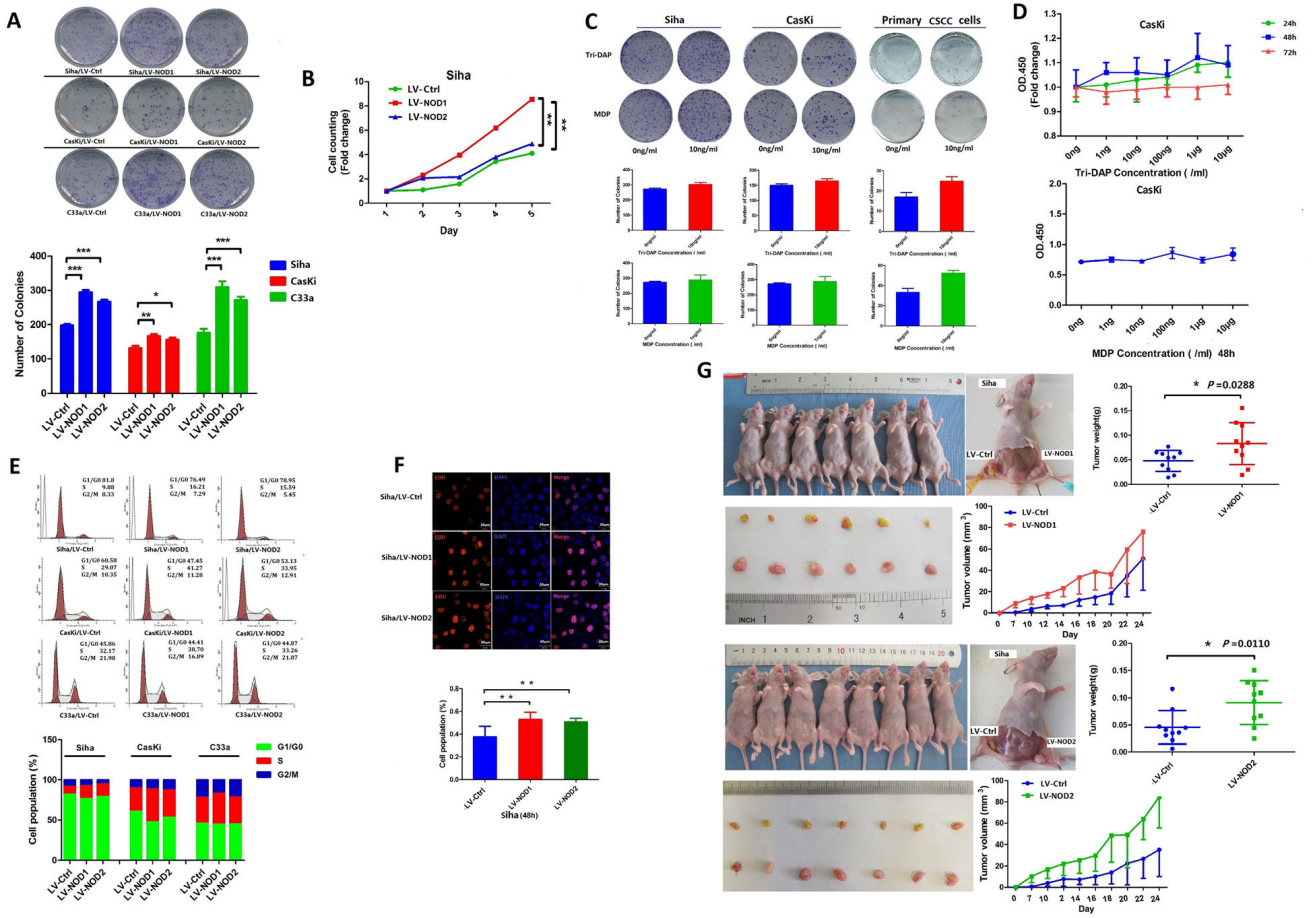
NOD1 and NOD2 enhanced the proliferation of CSCC cells. **A** Number of colonies formed by Siha, CasKi, and C33a cells overexpressing NOD1 or NOD2 (each group was performed triplicate wells, reproducible in three independent experiments). **B** Time-dependent increase in the number of Siha/LV-NOD1 and Siha/LV-NOD2 cells (results were highly reproducible triplicate: representative data of triplicate). **C** Number of colonies formed by cell lines and primary CSCC cells treated with Trip-DAP or MDP (10 ng/mL). **D** Proliferation of cell lines treated with various concentrations of NOD1 or NOD2 ligands. The assay of **C** and **D** was repeated thrice for the cell lines and twice for the primary cells using three independent samples. **E** Cell cycle distribution of control and NOD1/NOD2 overexpressing Siha, CasKi, and C33a cells. The percentage of cells in each phase is shown on the right. **F** Representative images of EDU-stained Siha/LV-NOD1, Siha/LV-NOD2, and Siha/LV-Ctrl cells (scale bar—30 μm, triplicate independent experiments). **G** Representative images of tumors in BALB/C nude mice subcutaneously injected with Siha/LV-NOD1, Siha/LV-NOD2 (right), and respective control cells (left). The tumor volume and weight of the indicated groups are shown on the right. All data are presented as mean ± SD (*n* = 10 for each group). *** *P* < 0.001; ** *P* < 0.01; * *P* < 0.05

The primary cells were purified and identified as previously described (Additional file 2: Fig.S2A). NOD1 and NOD2 were stably upregulated in the primary cells and cell lines with Tri-DAP and MDP stimulation, respectively (Additional file 2: Fig.S2B). In addition, the cell lines were transduced with NOD1/2-overexpressing lentiviruses (Additional file 2: Fig.S2C). Ectopic expression of NOD1 or NOD2 significantly enhanced the colony-forming potential of the CSCC cell lines (Fig. 2A, *P* < 0.05), which was consistent with their increased viability and proliferation rates in Siha cells (Fig. 2B). On the other hand, the NOD1 and NOD2 ligands only slightly enhanced the proliferative capacity of the CSCC cells (Fig. 2C, D). Consistent with the above, a significantly higher proportion of Siha/LV-NOD1/NOD2 cells were observed in the S phase compared to the Siha/LV-ctrl cells (Fig. 2E, F; *P* < 0.01). Likewise, the Siha/LV-NOD1 and Siha/LV-NOD2 cells resulted in significantly larger tumors in vivo compared to the control cells (Fig. 2G).

**Fig. 3:**
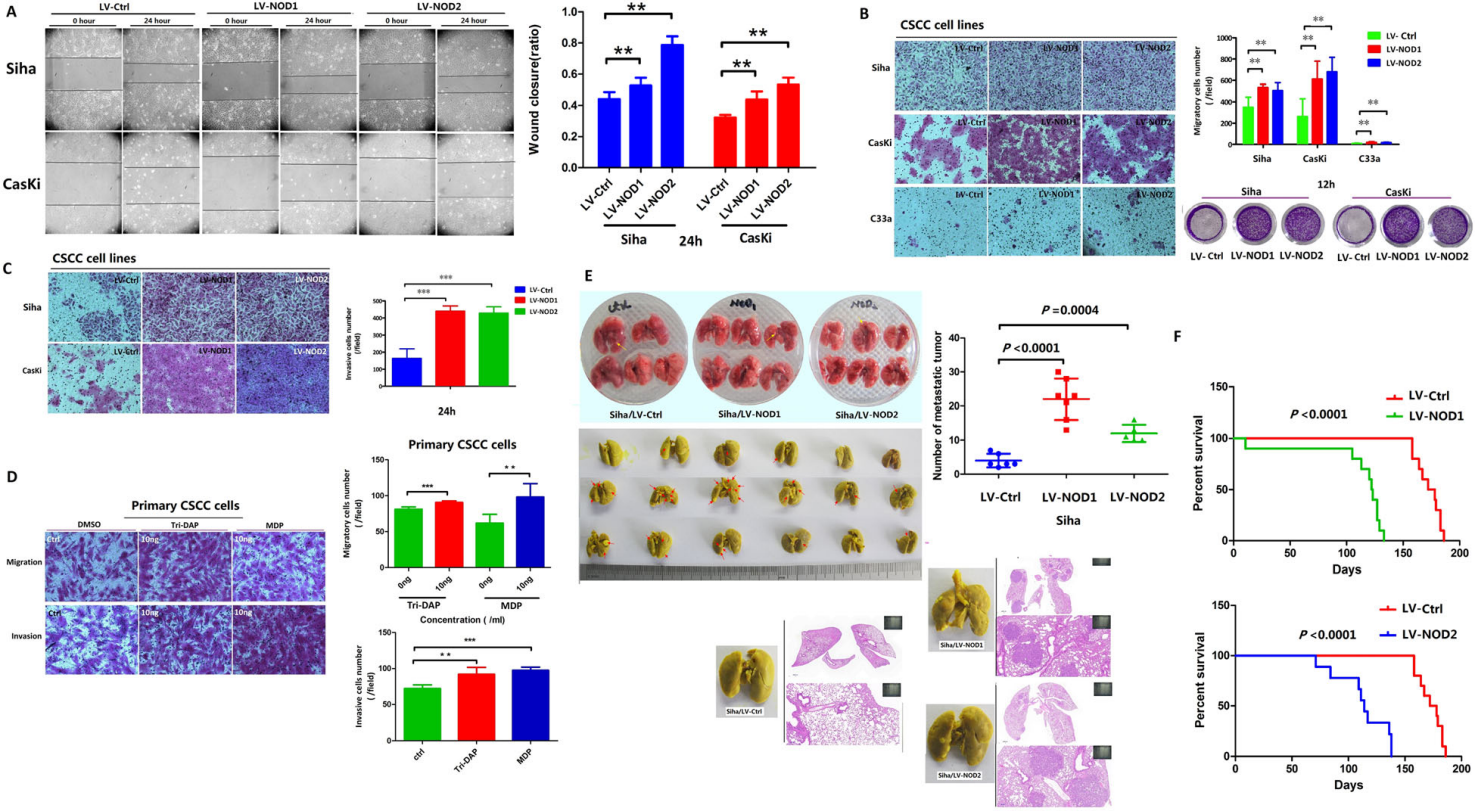
NOD1 and NOD2 promoted migration and invasion of CSCC cells. **A** Representative images showing wound closure of control and NOD1/2-overexpressing Siha and CasKi cells at 24 h after scratching. The percentage of wound healing is shown on the right. Data represent triplicates of one representative experiment. **B, C** The representative images indicating migration (panel **B**) (Siha, CasKi and C33a) and invasion (panel **C**) (Siha and CasKi) ability of NOD1/NOD2-overexpressing cell lines by transwell assay (reproducible in three independent experiments in triplicate wells). **D** Representative migration and invasion images of the primary CSCC cells by transwell members (The cells were pretreated with 10 ng/mL Tri-DAP or MDP at 24 h) (three independent experiments with three primary CSCC cells, triplicate wells; magnification × 100). **E** Number of lung metastatic nodules (The yellow arrows indicate representatively metastatic nodes in fresh lung; the red arrows indicate the nodes in fixed lung) in mice injected with Siha/LV-Ctrl, Siha/LV-NOD1, and Siha/LV-NOD2 cells (mouse harboring Siha/LV-Ctrl: *n* = 6, mouse harboring Siha/LV-NOD1: *n* = 6, mouse harboring Siha/LV-NOD2: *n* = 6). **F** Kaplan-Meier curves showing OS of the above tumor-bearing groups (*n* = 10 for each group). All data are presented as mean ± SD. *P < 0.05; ** *P* < 0.01; *** *P* < 0.001

The impact of NOD1 and NOD2 expression on the metastatic potential of CSCC cells was analyzed by in vitro and in vivo assays [28]. NOD1 and NOD2 overexpression significantly increased the extent of wound closure, as well the number of cells that migrated or invaded into the bottom surface of the transwell insert membranes (Fig. 3A–C, Additional file 3: Fig.S3A). The metastatic effect of NOD1/2 was also verified on the primary CSCC cells (Fig. 3D). Furthermore, the number of pulmonary metastatic nodules was markedly higher in the mice injected intravenously with Siha/LV-NOD1/NOD2 cells as opposed to the control Siha cells (Fig. 3E). Although the weight of the tumor-bearing mice was similar across the three groups (Additional file 3: Fig.S3B), the animals harboring Siha/LV-NOD1 or Siha/LV-NOD2 tumors had worse survival rates (Fig. 3F). Taken together, NOD1 and NOD2 significantly promoted CSCC proliferation by accelerating transition into the S phase of the cell cycle, and increased their metastatic potential.

### NOD1 and NOD2 enhanced the tumorigenic and metastatic potential of CSCC cells through multiple pathways

**Fig. 4:**
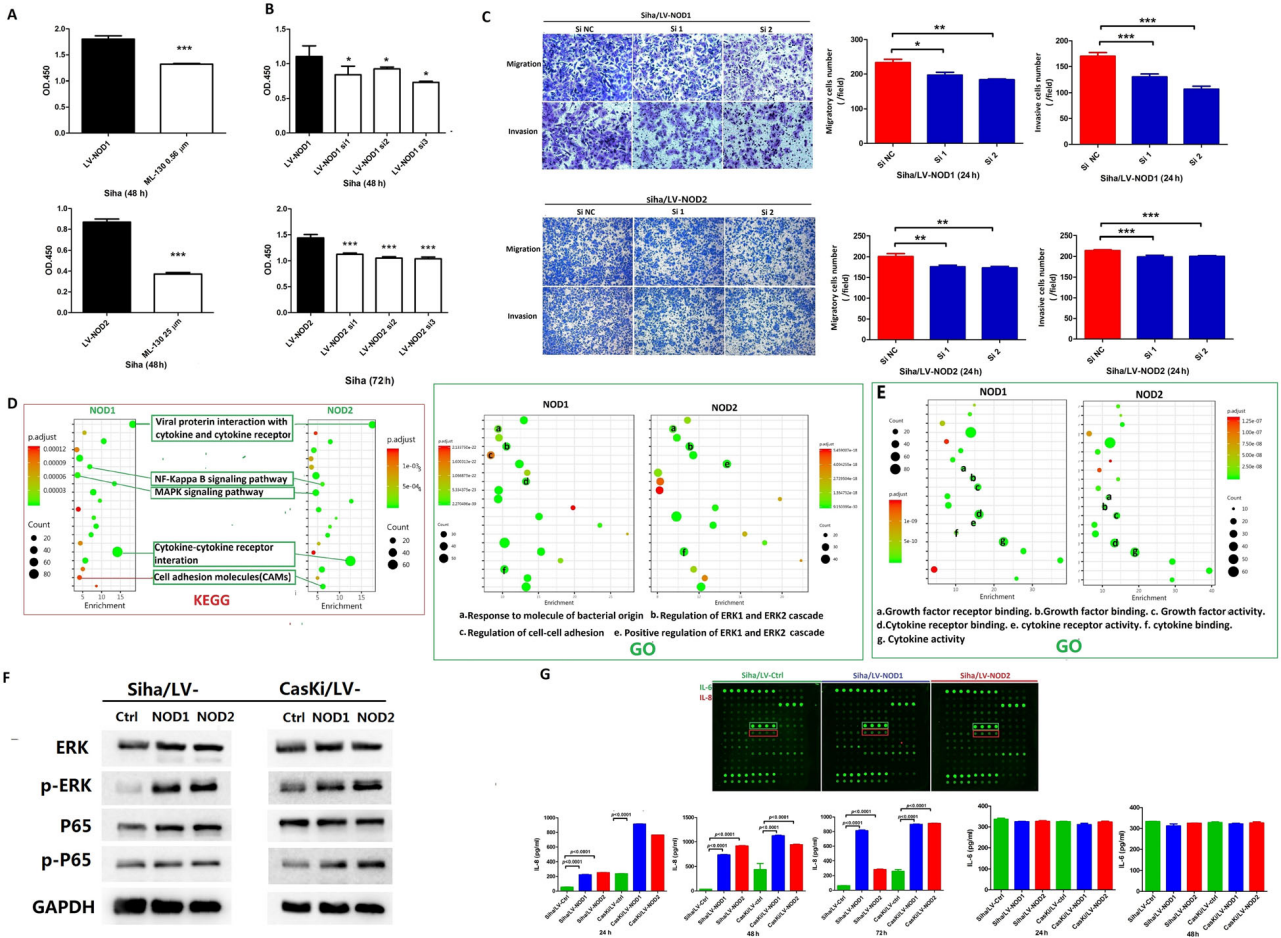
NOD1/NOD2 promote CSCC tumorigenesis by activating the ERK and NF-κB signaling pathways. **A** Proliferation rates of Siha/LV-NOD1 and Siha/LV-NOD2 treated with 0.56 μM (upper) and 25 μM (lower) ML-130. **B** Proliferation of Siha/LV-NOD1 and Siha/LV-NOD2 cells transfected with the NOD1 and NOD2 siRNAs. **C** Representative images of transwell assay showing the migration and invasion of Siha/LV-NOD1 and Siha/LV-NOD2 cells with NOD1 or NOD2 knockdown. **D** Protein arrays showing the molecules and signaling pathways involved in the upregulation of NOD1 and NOD2 (data was merged with Siha and CasKi cells)—viral protein interactions (left), bacterial response (right), MAPK, and NF-κB signaling pathways (left) and ERK cascade (right). **E** GO analysis showing enriched proteins in Siha and CasKi cells transfected with LV-NOD1 or LV-NOD2 compared to LV-Ctrl. **F** Immunoblots showing expression of ERK/p-ERK and NF-κB/p-NF-κB in LV-NOD1 or LV-NOD2 CSCC cell lines (Siha and CasKi). **G** Representative cytokine array panels for Siha/LV-NOD1 and Siha/LV-NOD2 cells (upper panel) and cytokine levels validated by ELISA (lower panel). Data of CSCC cell lines represent triplicate independent experiment of representative one. The data of **C** and **G** were performed with triplicate wells. All data are presented as the mean ± SD. *P < 0.05; **P < 0.01; ***P < 0.001

**Fig. 5:**
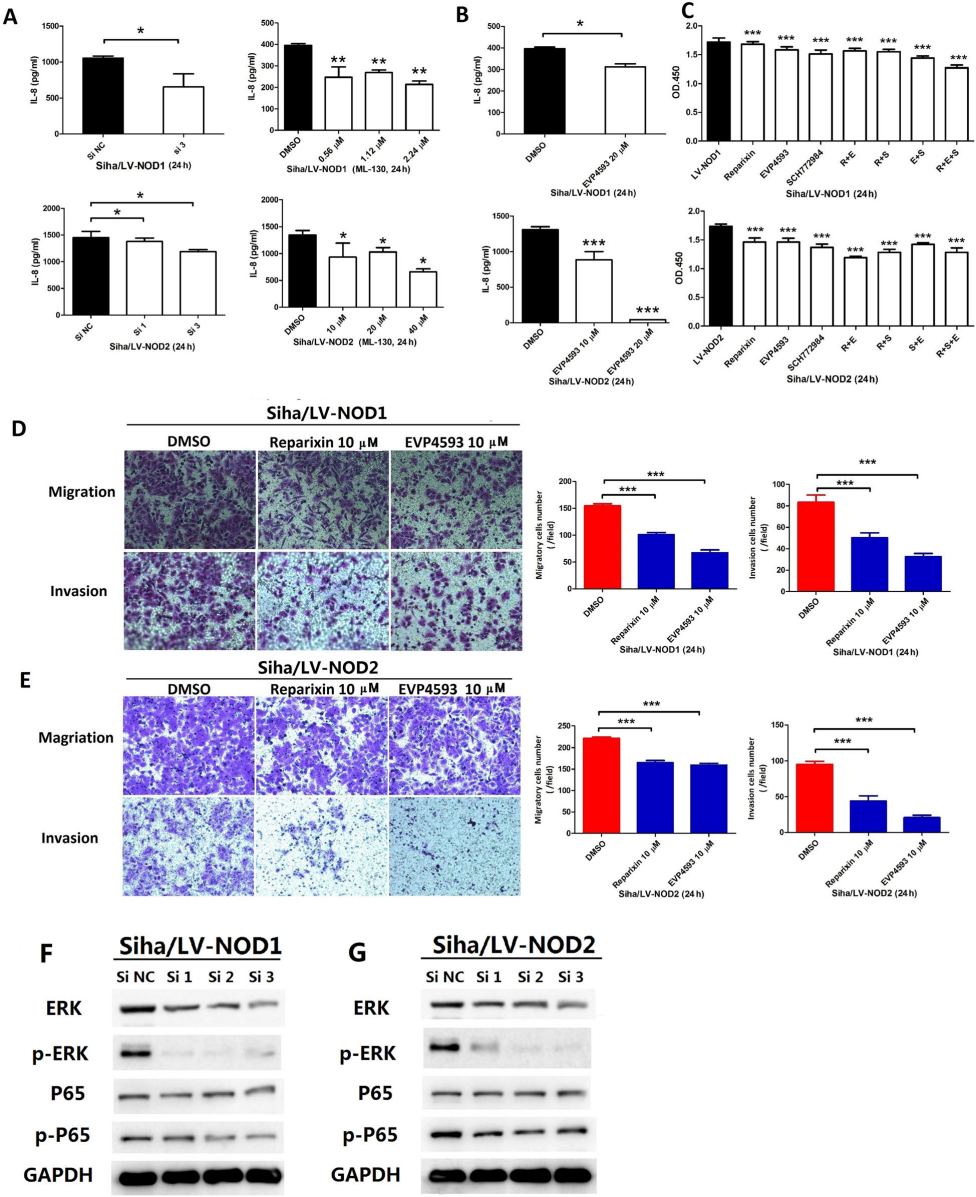
Reparixin inhibits NOD1/2-induced tumorigenesis of CSCC cells by inhibiting the secretion of IL-8. **A** IL-8 levels secreted by Siha/LV-NOD1 and Siha/LV-NOD2 cells with NOD1 and NOD2 knockdown (left panels), or ML-130 pretreatment (right panels). **B** IL-8 levels secreted by Siha/LV-NOD1 and Siha/LV-NOD2 treated with EVP4593 (NF-κB inhibitor). **C** Proliferation rates of Siha/LV-NOD1 and Siha/LV-NOD2 cells treated with Reparixin (R), EVP4593 (E), SCH772984 (S), and their combination. **D, E** Representative images of transwell assays showing migration and invasion of Siha/LV-NOD1 and Siha/LV-NOD2 cells treated with Reparixin or EVP4593. The percentages are shown on the right. ELISA and transwell assays were performed in triplicates and CCK8 assay in five replicates. **F, G** Immunoblot showing expression of ERK/p-ERK and NF-κB/p-NF-κB in the Siha/LV-NOD1 and Siha/LV-NOD2 CSCC cells (data are from three independent experiment) transfected with siRNAs. All data are presented as mean ± SD. *P < 0.05; **P < 0.01; ***P < 0.001

The mechanisms underlying the oncogenic effects of NOD1/NOD2 were further elucidated via pharmacological inhibition with ML-130 as well as siRNA-mediated gene knockdown (Additional file 4: Fig.S4). Inhibition of NOD1 or NOD2 significantly decreased the proliferative capacity (Fig. 4A, B), and the migration and invasion rates of Siha/LV-NOD1 or Siha/LV-NOD2 respectively (Fig. 4C) compared to the vehicle controls. GO and KEGG enrichment analyses further showed that the genes and proteins correlated with the upregulation of NOD1 or NOD2 were involved in cell proliferation, cytokines, and pathways in cancer such as ERK, NF-κB, and IL-8 (Fig. 4D–G). As shown in Fig. 4F, the ERK and NF-κB pathway proteins were also upregulated in the LV-NOD1 and LV-NOD2 cells. The quantity of cytokine in-cell array indicated that IL-6 and IL-8 were upregulated by NOD1 or NOD2 increasing (Fig. 4G upper panels); however, only significant higher IL-8 secretion by Siha/LV-NOD1 or Siha/LV-NOD2 was identified (Fig. 4G, down panels). NOD1/NOD2 increased the secretion of IL-8 but not of IL-6 (Fig. 4G, down panels), which was abrogated by their respective siRNAs (Fig. 5A, left panels) as well as ML-130 (Fig. 5A, right panels) and the NF-κB inhibitor (Fig. 5B). In addition, the proliferation ability of Siha/LV-NOD1 or Siha/LV-NOD2 was significantly inhibited by selective inhibitors of the IL-8 receptor CXCR1/2 (Reparixin), NF-κB (EVP4593), or ERK (SCH772984) (Fig. 5C), and the combination of all three showed a cumulative inhibitory effect (Fig. 5C). Reparixin and EVP4593 also decreased the metastatic potential of Siha/LV-NOD1 and Siha/LV-NOD2 cells (Fig. 5D, E).

The enhanced IL-8 secretion by NOD1/NOD2-overexpressing Siha cells upregulated the adhesion molecule FN1 (Additional file 5: Fig.S5A), and knocking down FN1 inhibited metastasis of Siha/LV-NOD1 and Siha/LV-NOD2 cells (Additional file 5: Fig.S5B). In addition, the elevated FN1 in Siha/LV-NOD1 or Siha/LV-NOD2 cells was downregulated by Reparixin (Additional file 5C). To summarize, the oncogenic effects of NOD1 and NOD2 in CSCC are mediated through multiple pathways; and knocking down either NOD1 or NOD2 in Siha cells downregulated P65/p-P65and ERK/p-ERK significantly (Fig. 5F, G).

### Reparixin prolonged the survival of mice harboring NOD1^high^ tumors

**Fig. 6:**
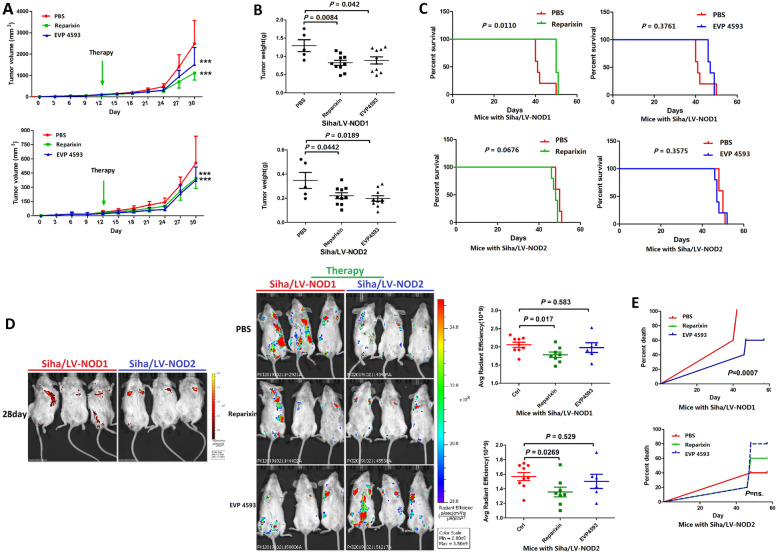
Reparixin inhibited growth of NOD1^high^ and NOD2^high^ CSCC tumors ***in vivo***. **A** Volume of Siha/LV-NOD1 and Siha/LV-NOD2 tumors in the untreated (*n* = 5), Reparixin (*n* = 10), and EVP4593 (*n* = 10) groups. **B** Tumor weight in the Reparixin (*n* = 10), EVP4593 (*n* = 10), and PBS (*n* = 5) groups. **C** OS Kaplan-Meier curves of mice in the above groups. **D** Representative fluorescence images showing metastasis of GFP+ Siha/LV-NOD1 or Siha/LV-NOD2 cells in the indicated groups (mouse harboring GFP+ Siha/LV-NOD1: Group treated by PBS: *n* = 9; group treated by Reparixin: *n* = 8; group treated by EVP4593: *n* = 6. mouse harboring GFP+ Siha/LV-NOD2: Group treated by PBS: *n* = 9; group treated by Reparixin: *n* = 8; group treated by EVP4593: *n* = 6). Quantification of GFP intensity is shown on the right. **E** Survival curves of the above tumor-bearing mice treated with PBS, Reparixin, or EVP4593. The results are presented as mean ± SD. ***P < 0.001; **P < 0.01; * *P* < 0.05

Consistent with the in vitro findings, Reparixin or EVP4593 markedly decreased the volume and weight (Fig. 6A, B) of tumors derived from mice with subcutaneous Siha/LV-NOD1 or Siha/LV-NOD2 cells compared to the untreated controls. Reparixin significantly improved the OS of mice bearing Siha/LV-NOD1 tumors subcutaneously (Fig. 6C). In the metastasis models induced by tail vein injection with Siha/LV-NOD1 or Siha/LV-NOD2 cells, Reparixin inhibited the growth of metastatic nodules compared to the placebo controls (Fig. 6D). Finally, Reparixin significantly improved the OS of mice with metastatic Siha/LV-NOD1 nodule xenografts (Fig. 6E). Taken together, the NOD1/ NF-κB/IL-8 axis is a promising therapeutic target in CSCC.

## Discussion

Metastatic and recurrent CSCC are highly recalcitrant tumors and challenging to treat. Previous study indicated several PRRs have been implicated in the progression of cervical cancer [23], the NLR family of PRRs has been identified in host immune defense [11, 29], and its members NOD1 and NOD2 are widely expressed in the female reproductive organs including endometrium, fallopian tubes, cervix, and ecto-cervix [27, 30, 31]. NOD1 plays an important role in the development of colon cancer and breast cancer [11, 20, 32, 33], and its dysregulation drives the progression of CIN to cervical cancer [27]. The correlation between NOD2 expression and tumorigenesis varies across different cancer types [33–36]. We detected higher levels of NOD1 and NOD2 in the CSCC tissues compared to the normal cervix. Furthermore, NOD1 was particularly overexpressed in tumors with LVSI, LM, and poor differentiation and associated with worse survival. NOD2 was elevated in the tumors with LVSI and poor differentiation, although its association with LM and survival was not as significant as observed with NOD1. In clinical characteristics, LVSI and LM associate with higher metastatic rate. The higher risk for worse prognosis is LM, and the Sedlis criteria include risk factor of LVSI for worse prognosis [37]. Our results also indicated that higher NOD1 or NOD2 expression was not associated with advanced tumor stages. As we know, advanced stage cervical cancer means worse OS; poor differentiation is not a definitely middle/high-risk factor for prognosis in patients with CSCC [38]. From the above results, we can predict that CSCC patients in the same stage with the higher NOD1 expression have worse prognosis. However, we collected the CSCC tissue from “Oct 2017 to Dec 2019” and most of these samples were from patients at early stages (IB1-IIA, FIGO 2009 staging system), and we could not get the powerful survival data from our enrolled patients (OS and DFS (disease free survival)) because of the relatively short follow-up period. Therefore, we abstracted the clinical characteristics from “TCGA” database and calculated the OS.

Furthermore, in a functional experiment, the ectopic expression of NOD1 or NOD2 in CSCC cell lines enhanced their proliferative and metastatic capacities in vitro and in vivo. Besides, we isolated and cultured primary CSCC cells for counterpart experiments of CSCC cell lines since there is a limited source of cell lines in the world. The upregulation of NOD1 in primary CSCC cells also increased the metastatic capacity in vitro as cell lines showed. This is consistent with the increased expression of NOD1 observed in colon cancer metastasis and breast cancer cell lines [33, 39]. However, Liu et al reported a suppressive role of NOD1 in CSCC [27], which might point to a differential function depending on the disease stage.

NOD1 and NOD2 stimulation by their respective ligand activates the ERK and NF-κB signaling pathways [33, 40, 41], and several studies have demonstrated NOD1/2-mediated phosphorylation of ERK and P65 [11, 12, 33, 36, 42, 43]. The activation of NF-κB and ERK pathways culminates in the upregulation in multiple downstream targets, such as IL-8 and fibronectin (FN1) [44–47]. FN1 is extravasated from the bloodstream into tissues and promotes tumor adhesion [39, 48] in response to increased IL-8 secretion [49]. Both TLRs and NODs are involved in CSCC progression [50], and upregulation of TLRs (such as TLR8) may also induce IL8 secretion [51]. Additionally, previous studies have demonstrated that the IL-8-CXCR1/2 axis is involved in the tumorigenesis and metastasis of multiple cancers [44, 52, 53] and the safety of ML-130 has not been examined by any clinical trial; we surmised that NF-κB/IL-8 are potential therapeutic targets in CSCC patients with metastasis.

Indeed, tumor progression was remarkably attenuated by the CXCR1/2 inhibitor Reparixin, whereas inhibition of NF-κB had limited effect given the involvement of multiple signaling pathways. Consistent with our findings, IL-8 and CXCR1/2 inhibitors significantly attenuated progression of breast cancer [54–56]. Anti-IL-8 treatment regimens are currently in the clinical testing phase for non-small cell lung cancer (NSCLC), hepatocellular carcinoma (HCC) (NCT04123379, recruiting), and early (NCT01861054) and metastatic breast cancer (NCT02001974). Interestingly, Reparixin significantly improved the survival of mice bearing metastatic Siha/LV-NOD1 tumors but its therapeutic effect was less pronounced in the Siha/LV-NOD2 group.

## Conclusions

In summary, NOD1 is a potential biomarker of worse prognosis in CSCC patients. Both NOD1 and NOD2 enhanced the proliferative and metastatic abilities of CSCC cells by promoting IL-8 secretion via the NF-κB pathways. Furthermore, Reparixin is a promising agent against CSCC and should be further examined in a clinical setting in NOD1-positive populations with metastatic CSCC.

## Methods

### Patient samples and clinicopathological data

Specimen collection and clinicopathological data review were approved by the Ethics Committee of Cancer Hospital, CAMS (Chinese Academy of Medical Sciences & Peking Union Medical College). This study was performed in accordance with the International Ethical Guidelines for Biomedical Research Involving Human Subjects (CIOMS), and none of the procedures conducted in this study interfered with the treatment plan of the patients. CSCC tissues were collected during surgery or biopsies conducted between Oct 2017 and Dec 2019 at the Cancer Hospital, after obtaining consent from the patients. Totally, fifty-eight CSCC samples and thirty-three normal cervix tissue samples were used for the quantitative real-time PCR (qPCR) assay, and 143 CSCC samples were collected for immunohistochemistry (IHC). Sixteen tumor samples were used for primary cell isolation and subsequent assays.

### Transcriptome sequencing and bioinformatics analysis

The NOD1 and NOD2 expression data of CSCC patients was extracted from the Human Protein Atlas (http://www.proteinatlas.org), and the mRNA expression and survival data from The Cancer Genome Atlas (TCGA) databases. The transcriptome sequencing (differential expression genes, DEG) of CSCC and normal cervical tissue (from Cancer Hospital) were identified by BGISEQ platform and analyzed on the DR. TOM network platform of BGI (https://biosys.bgi.com/#/report/login). The sequencing reads which contain low-quality, adaptor-polluted, and high content of unknown base (N) reads should be processed to be removed before downstream analyses. After sequencing data filtering, DEG level was calculated for each sample with RSEM [57]. The target genes were functionally annotated by Gene Ontology (GO) and Kyoto Encyclopedia of Genes and Genomes (KEGG) pathway analyses, and the significant biological processes, cellular components, and molecular functions were identified.

### Cell culture

The human CSCC cell lines Siha, CasKi, and C33a were all purchased from Cell Resource Center (Beijing, China). The cell lines were verified by short tandem repeat (STR) sequencing by the Beijing Microread Genetics Company on July 2018. The cells were cultured in DMEM/F12 medium (Lonza, Walkersville, MD, USA) supplemented with 10% fetal bovine serum (FBS) (Gibco, Thermo Fisher Scientific, USA) and 1% penicillin/streptomycin (PS) (Gibco, Thermo Fisher Scientific, USA) at 37 °C under 5% CO_2_ (Thermo Technologies, Vancouver, Japan). The cells were treated with Tri-DAP, MDP (InvivoGen, USA), ML-130 (TargetMol, USA), CXCR1/2 inhibitor (Reparixin, Med Chem Express, USA), NF-κB inhibitor (EVP4593, Sellect, USA), or ERK inhibitor (SCH772984, Sellect, USA) as required. Primary CSCC cells were isolated from patient samples as previously described [58]. Briefly, the specimens were minced into 1-mm^3^ pieces in 6-cm petri dishes, and sequentially digested with 0.05% trypsin containing EDTA (Lonza, Walkersville, MD, USA) and 0.2% type I collagenase (Sigma-Aldrich Corp., St Louis, MO, USA) at 37 °C with constant shaking. FBS was added to terminate the reaction, and the cells were washed and re-suspended in DMEM/F12 complete medium with 5% FBS (Gibco). The primary cells were seeded in a petri dish and cultured for 7–10 days.

### MACS and flow cytometry

Trypsinized primary CSCC cells were re-suspended in MACS (magnetic-activated cell sorting) separation buffer and incubated with anti-EpCAM magnetic microbeads (Miltenyi Biotec Inc, Auburn, CA, USA) according to the manufacturer’s instructions. Then, the EpCAM-positive cells were collected and cultured in high-glucose DMEM medium with FBS (5%). Flow cytometry (FCM) was used to identify the purity of the CSCCs immediately after cell sorting or a short period of culture: Purified primary cells were stained with fluorescent-conjugated antibodies against anti-human EpCAM FITC (BioLegend Inc., San Diego, CA, USA) and anti-human vimentin PE (Miltenyi Biotec Inc, Auburn, CA, USA) on ice. Since vimentin was the cellular marker used, the cells were pretreated with Fixation/Permeabilization reagent (Invitrogen, Carlsbad, CA) according to the protocols recommended by the manufacturer. FCM acquisition was performed using a Beckman coulter-Dxflex flow cytometer. Flow Jo V10 software was used for the data analysis. The purity of the sorted cells reached ∽ 96% purity. After the purification was identified, the cells were collected and cultured for functional examination.

### Quantitative real-time PCR

Total RNA was extracted from the cultured cells and frozen tissues using Trizol Reagent (Invitrogen, Carlsbad, CA). The quality and concentration of the RNA were determined using a Nanodrop Spectrophotometer (Thermo Scientific, Wilmington, DE). The RNA was reverse transcribed to cDNA using a Reverse Transcriptase Kit (Takara, Japan) and amplified by RT-qPCR using Power SYBR Green PCR Master Mix (Life, Applied Biosystems) on the Step One Plus Real-Time PCR System (Life, Applied Biosystems). The target gene expression levels were calculated with the 2^−ΔΔCt^ method, and each sample was analyzed in triplicates. The primers were synthesized by Sangon Technologies (Shanghai, Corp.) and the sequences were as follows:

- NOD1:FW5′-TACTGAAAAGCAATCGGGAACT,
- RW: 5′-GTAGAGGAAGAACTCGGACACC;
- NOD2: FW: 5′-TGCGGACTCTACTCTTTGAGC,
- RW: 5′-CCGTGAACCTGAACTTGAACT;
- GAPDH: FW: 5′-GCACCGTCAAGGCTGAGAAC,
- RW: 5′-TGGTGAAGACGCCAGTGGA.

### Hematoxylin-eosin staining (HE) and immunohistochemistry (IHC)

The tissue samples were fixed with 4% paraformaldehyde for 24 h, embedded in paraffin, cut into 5-μm-thick sections, and coated at 75 °C for 2 h. The sections were deparaffinized using xylene and rehydrated through an ethanol gradient. HE staining was performed as standard protocols. For IHC, the sections were heated in citrate buffer for antigen retrieval, incubated with 2% hydrogen peroxide for quenching endogenous peroxidase, and blocked using 1% goat serum (ZSJQ, Beijing, China). The slices were then incubated overnight with rabbit anti-human vimentin (without diluted, Origene, Beijing, China), mouse anti-human pan-cytokeratin AE1/AE3 (without diluted, Origene, Beijing, China), mouse anti-human Ki67 (diluted 1:200, ZSGB-BIO, Beijing, China), mouse anti-human P16 (diluted 1:200, ZSGB-BIO, Beijing, China), mouse anti-human NOD1 (B-4; dilution: 1:100; sc-398696, Santa Cruz, USA), and mouse anti-human NOD2 (2D9; dilution: 1:100; sc-56168, Santa Cruz, USA) antibodies and the isotype control at 4 °C. After washing with PBST (Phosphate Buffer Solution with Tween-20), the sections were sequentially stained with DAB chromogen (diaminobezidin, ZSJQ, Beijing, China) and hematoxylin (Sigma-Aldrich, USA). NOD1 and NOD2 expression parameters were scored by Image Pro Plus software (USA).

### Immunofluorescence staining

The cells cultured on chamber slides were fixed with cold 4% paraformaldehyde and permeabilized with 0.1% Triton X-100 for 15 min. After washing with PBS, the cells were incubated overnight with rabbit anti-human vimentin (diluted 1:100, Abcam, Cambridge, MA, USA), mouse anti-human AE1/AE3 (diluted 1:100, ZSGB-BIO, Beijing, China), rabbit anti-human CDKN2A/p16INK4a (P16) (diluted 1:200, Abcam), mouse anti-human NOD1 (B-4; dilution: 1:100; sc-398696, Santa Cruz), and mouse anti-human NOD2 (2D9; dilution: 1:100; sc-56168, Santa Cruz) primary antibodies. The slides were then incubated with Alexa Fluor-conjugated secondary antibodies (Abcam) for 1 h at room temperature and counterstained with DAPI (4′,6-diamidino-2-phenylindole, Cat.H3570, Life Technologies). The stained tissues and cells were viewed using Olympus scanner or laser scanning confocal microscope (LSM780; Zeiss), and the staining intensity was evaluated by a pathologist blinded to the samples using image plus software (USA).

### Lentiviral transduction

Human NOD1 (BC040339.1) and NOD2 (NM022162.2) were respectively cloned into the pLVX-P2A-ZsGreen-T2A-Puro vector at the XhoI and BamHI sites. The lentivirus with pLVX-hNOD1/hNOD2-ZsGreen-Puro was purchased and packaged as per the manufacturer’s description (Likeli Biotec Inc, Beijing, China USA). The CSCC cell lines (Siha, CasKi and C33a) were transduced with the NOD1/NOD2 or empty vector lentiviruses and selected using puromycin. NOD1/2 overexpression was verified by western blotting.

### Small interfering RNA (siRNA) transfection

NOD1, NOD2, FN1, and scrambled siRNAs were synthesized by JTS Scientific Company (Beijing, China). The sequences are as follows:

- NOD1: Si1 (866) - CCUGCUCACUCAGAGCAAAtt, UUUGCUCUGAGUGAGCAGGtt
- Si2 (1240) - GCAUGUUCAGCUGCUUCAAtt, UUGAAGCAGCUGAACAUGCtt
- Si3 (2095) - CCUUCUUUACAGCCUUCUUtt, AAGAAGGCUGUAAAGAAGGtt
- NOD2: Si1 (952) - GCAAGAAGUAUAUGGCCAAtt, UUGGCCAUAUACUUCUUGCtt
- Si2 (1253) - GCAAGACUUCCAGGAAUUUtt, AAAUUCCUGGAAGUCUUGCtt
- Si3 (2798) - GCUCAUUGAAUGUGCUCUUtt, AAGAGCACAUUCAAUGAGCtt
- FN1 - CCAUUUCACCUUCAGACAAtt, UUGUCUGAAGGUGAAAUGGtt

The Siha/LV-NOD1, Siha/LV-NOD2, CasKi/LV-NOD1, and CasKi /LV-NOD2 cell lines were grown till 70% confluency and transfected with the respective siRNAs using Lipofectamine™ 2000 (Invitrogen, Thermo Fisher Scientific). Briefly, siRNA and 1 μl Lipofectamine™ 2000 was respectively diluted in 50 μl Opti-MEM, incubated for 15 min at room temperature, and then mixed. The mixture was incubated for 15 min at room temperature and added to each well. The cells were incubated at 37 °C for 24 h, and the transfection efficacy was tested.

### Human cytokine array, western blotting, and ELISA

The cultured cells were harvested for cytokine array and western blotting, and the supernatants were collected for ELISA. The cytokine levels were analyzed with the G-Series Human Cytokine Antibody Array 440 as per the manufacturer’s instructions (Ray Biotech Inc. Quantibody service, China). Western blotting was performed using standard protocols after the cells were lysed in RIPA buffer (Sigma, Saint Louis, MO) [59]. The following primary antibodies were used: β-actin (AC-15; 1:2000; Sigma), NOD1 (mouse anti-human, B-4; 1:500; sc-398696, Santa Cruz), NOD2 (mouse anti-human, 2D9; 1:500; sc-56168, Santa Cruz), P65 (rabbit anti-human, D14E12, 1:1000; CST, USA), p-P65 (rabbit anti-human, 93H1, 1:1000; CST, USA), P44/42 MAPK (ERK1/2) (rabbit anti-human, 1:1000; CST, USA), and pP44/42 MAPK (pERK1/2) (Thr202/Tyr204, rabbit anti-human, 1:1000, CST, USA). The IL-8 and IL-6 levels in the supernatants were quantified using LEGEND MAX™ Human IL-8 and Human IL-6 ELISA Kits (Biolegend, USA) according to the manufacturer’s instructions.

### Cell counting and CCK8 assays

Cells were seeded in 6-cm petri dishes at the logarithmic phase of growth, and harvested after 24, 48, 72, 96, and 120 h, respectively. The number of cells was recorded using a cell counter II (Life Corp, USA). The cells were seeded into 96-well plates for the Cell Counting Kit-8 (CCK8) assay (Dojindo Laboratories, Japan), and 10 μl CCK8 solution was added per well at 24, 48, and 72 h of culture. The optical density at 450 nm (OD.450) was measured using a Model 680 Microplate Reader (BIO-RAD, Hercules, CA). Five replicates were tested per sample.

### Cell cycle profiling

The transfected cells were seeded into 12-well plates and cultured for 24 h. After fixing in cold 4% paraformaldehyde (PFA) for 15 min and permeabilizing with 0.1% Triton X-100 for 15 min, the cells were incubated with EdU (5-Ethynyl-2'-deoxyuridine) (Beyotime Biotechnology, China) for proliferation. The cells were then washed thrice with PBS, counterstained with DAPI (Beyotime Biotechnology, China), and observed under a laser scanning confocal microscope (LSM780; Zeiss). For FCM, the cells were harvested, washed sequentially with citrate buffer and PBS, and incubated with ribonuclease (RNAase) and propidium iodide (PI) (Ref. CYT-PIR-25, Cytognos, Spain) at room temperature for 1 h. The stained cells were acquired in a flow cytometer (Beckman coulter-Dxflex) and analyzed by the Flow Jo V10 software.

### Colony formation assay

The primary cells and cell lines were seeded in six-well plates at the respective densities of 800 cells/well and 500 cells/well. After culturing for 10–14 days, the cells were fixed with cold 4% PFA and stained with crystal violet (Solarbio, Beijing, China).

### Wound healing assay

Siha/LV-Ctrl/NOD1/NOD2 and CasKi/LV-Ctrl/NOD1/NOD2 cells were seeded into 6-well plates at the density of 1 × 10^6^ cells/well in complete DMEM/F12 (10% FBS). After 24 h of culture, the monolayer was scratched using a 10-μl pipette tip, and the wound region was measured at 0, 6, 12, and 24 h under a microscope. The wound healing rate at the different time points was quantified as the width of the wound region relative to the initial width at 0 h.

### Transwell assay

The different cell lines and primary cells were seeded into transwell chambers (Costar, Cambridge, MA) at the respective densities of 3–5 × 10^4^ cells/well and 0.5–1 × 10^5^ cells/well in 200 μl serum-free DMEM/F12. The lower chambers were filled with 600 μl complete DMEM/F12 (10% FBS). After 20–24 h of incubation, the cells that had migrated through the membrane were fixed and stained, and counted in four randomly chosen fields. The invasive capacity of the cells was similarly analyzed using Matrigel-coated (BD Biosciences, San Jose, CA, USA) transwell membranes.

### In vivo experiments

All animal experiments were approved by the Beijing Municipal Science and Technology Commission, and conducted in accordance with the relevant guidelines. The xenograft model was established by subcutaneously inoculating 8-week-old BALB/c nude mice or 7–8 weeks NOD/SCID mice with 3 × 10^6^ Siha/LV-Ctrl/NOD1/NOD2 cells and bilaterally. Palpable tumors (> 3 mm) appeared 7 days after injection and were measured every 3 days. The mice were euthanized 28–56 days post-inoculation, and the tumors were removed and weighed. The lung metastasis model was established by intravenously injecting 8–9-week-old NOD/SCID mice with 1 × 10^6^ Siha/LV-Ctrl/NOD1/NOD2 cells. Metastatic growth in the lungs was detected by labeling with luciferase or GEP. The mice were sacrificed 56–84 days, and the number of metastatic nodules was counted. For the treatment regimen, the tumor-bearing mice were divided into the placebo control, Reparixin, and EVP-4395 groups, and the tumor growth was monitored as described above.

### Statistical analysis

Statistical analysis was performed using SPSS 19.0 software, and GraphPad Prism 5.0 software was used for plotting graphs. Quantitative variables between two groups were compared by Student’s *t* test (normal distribution) or Mann-Whitney *U* test (non-normal distribution), and one-way or two-way ANOVA was used for comparing multiple groups. Pearson *χ*^2^ test or Fisher’s exact test was used to compare qualitative variables. Survival curves were plotted by the Kaplan-Meier method and compared by the log-rank test. *P* values of < 0.05 were considered statistically significant.

## Supplementary Information

**Additional file 1. **The IHC scores of NOD1 and NOD2 expression levels. A) Representative images of HE-stained human CSCC tissues (n = 113, magnification 10╳), P16 and/or Ki67 and/or CK immunostaining, and AE1/AE3 and vimentin immunofluorescence (*n* = 6; scale bar - 30 μm). B) DEGs by Venn diagrams (left panel): the red number represents the up-regulated gene amount, blue number represents the downregulated gene amount; Scatter plot (right panel): hierarchical clustering of 5,140 upregulated mRNAs, using X Y axis represents log10 transformed gene expression level, red color represents the up-regulated genes, blue color represents the downregulated genes, gray color represents the non-DEGs (Normal cervix, *n* = 4; cervical cancer, *n* = 6). C) IHC scores for NOD1 and NOD2 in the early and advanced stage tumors (for NOD1, I-II stages: n = 39, III-IV stages: *n* = 53; for NOD2, I-II stages: *n* = 34, III-IV stages: n = 52). D) IHC scores of NOD1 and NOD2 in tumors of different grades (for NOD1, high and middle: *n* = 46, poor: *n* = 60; for NOD2, high and middle: *n* = 43, poor: *n* = 50). E) The positive correlation mRNA expression of NOD1 and NOD2 was identified by database (*n* = 306, http://timer.cistrome.org). F) Kaplan–Meier curves showing overall survival of CSCC patients demarcated on the basis of in situ NOD1 and NOD2 expression (http://www.proteinatlas.org). All data are presented as mean ± SD. *, *P* < 0.05; **, *P* < 0.01; ***, *P* < 0.001.

**Additional file 2.** NOD1/2 expression in primary CSCC cells and CSCC cell lines. A) Morphology of the cultured Siha, Caski, C33a cell lines and the primary CSCC cells (left). EpCAM positive cells were sorted by MACS and identified by FCM (middle). The continuum of cultured primary CSCC cells were confirmed using immunofluorescence (AE1/AE3+ and P16+ and vimentin) (right). B) The level of NOD1 and NOD2 in CSCC cell lines and primary cells was upregulated through pretreatment by specific ligands (10 ng/mL) at 24 h by qPCR (upper) and in-cell immunofluorescence staining (lower). C) Stable NOD1 and NOD2 expression in the cultured Siha, CasKi and C33a cell lines were confirmed by green fluorescence (GFP flag), qPCR and western blotting.

**Additional file 3. **NOD1 and NOD2 enhanced the tumorigenic and metastatic abilities of CSCC cells. A) The percentages of wound healing, while data on C33a wound closure was normalized using the wound length at 0 h (left, 24 h and 48 h). The image on the right shows the percentage of wound closure of the Siha and CasKi cells, which were normalized to the wound length at 0 h (48 h). B) The weights of mice injected with different cell lines. ***, *P* < 0.001; **, *P* < 0.01; *, *P* < 0.05.

**Additional file 4.** Siha/LV-NOD1 and Siha/LV-NOD2 cells transfected by siRNA. The expression of NOD1 mRNA (A, data were from three independent experiments with three replicates) and protein (B, the picture is a representative from two independent experiments). SiRNA was presented as Si1, Si2 and Si3.

**Additional file 5.** Over-expression of NOD1 or NOD2 of Siha cells promotes FN1 and IL-8. A) Representative results of adhesion and invasion molecules with over-expression of NOD1/NOD2. (Siha cells, two independent experiments using triplicated wells). B) Transwell assays revealed that the migration and invasion abilities of the Siha/LV-NOD1 and Siha/LV-NOD2 cells were inhibited by knock down FN1. C) Reparixin downregulates FN1 mRNA expression.

## Data Availability

The NOD1 and NOD2 expression data of CSCC patients was extracted from the Human Protein Atlas (http://www.proteinatlas.org), and the mRNA expression and survival data from The Cancer Genome Atlas (TCGA) databases. The target genes were functionally annotated by Gene Ontology (GO) and KEGG pathway analyses, and the significant biological processes, cellular components, and molecular functions were identified. The primary experiment data and material can be obtained from the correspondence with reasonable require.

## Abbreviations

CSCC: Cervical squamous cell carcinoma
IL-8: Interleukin-8
IHC: Immunohistochemistry staining
qPCR: Quantitative real-time PCR
CI: Cell Index
mRNA: Messenger RNA
H&E staining: Hematoxylin-eosin staining
ELISA: Enzyme-linked immunosorbent assay
LVSI: Lympho-vascular space invasion
LM: Lymph nodes metastasis
FCM: Flow cytometry
HPV: Human papilloma virus
PRRs: Paternal recognition receptors
PD-1/ PDL-1: Program death-1/ program death ligand-1
NLRs: Nucleotide-binding oligomerization domain-like receptor
TLR: Toll like receptors
NOD: Nucleotide-binding oligomerization domain receptor
meso-DAP: Meso-diaminopimelic acid
MDP: Muramyl dipeptide
MACS: Magnetic-activated cell sorting
IF: Immunofluorescence
EdU: 5-Ethynyl-2'-deoxyuridine
PBS: Phosphate-buffered Saline
PFA: Paraformaldehyde
OS: Overall survival
CCR1-2: IL-8 receptor
