# Supplementary figures and images for: Upregulation of NOD1 and NOD2 contribute to cancer progression through the positive regulation of tumorigenicity and metastasis in human squamous cervical cancer
Source: BMC Med. 2022 Feb 8;20:55. doi: 10.1186/s12916-022-02248-w (PMC8822783; doi:10.1186/s12916-022-02248-w)

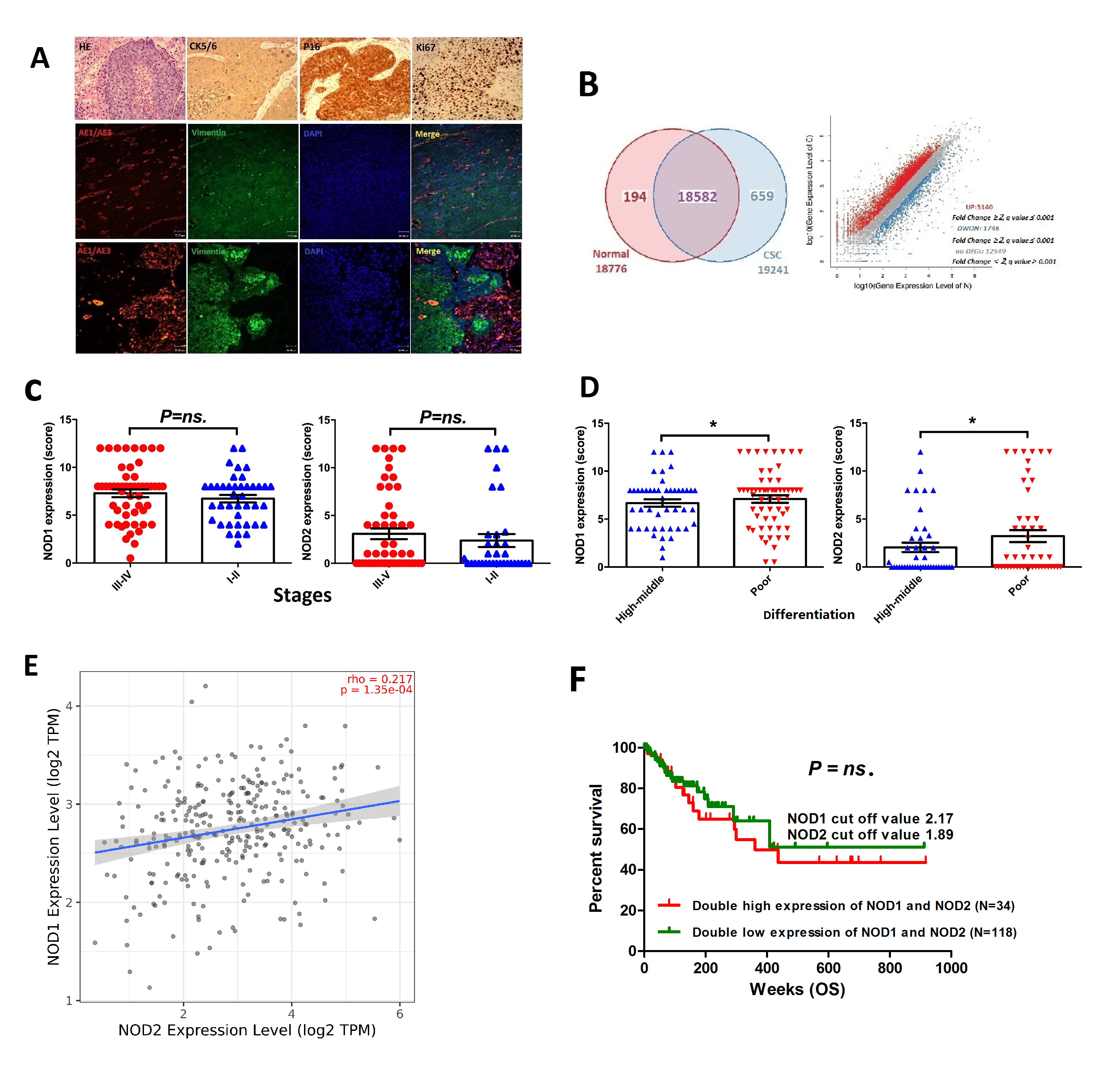

Supplement: Supplementary file 1 — Additional file 1. The IHC scores of NOD1 and NOD2 expression levels. A) Representative images of HE-stained human CSCC tissues (n = 113, magnification 10╳), P16 and/or Ki67 and/or CK immunostaining, and AE1/AE3 and vimentin immunofluorescence (n = 6; scale bar - 30 μm). B) DEGs by Venn diagrams (left panel): the red number represents the up-regulated gene amount, blue number represents the downregulated gene amount; Scatter plot (right panel): hierarchical clustering of 5,140 upregulated mRNAs, using X Y axis represents log10 transformed gene expression level, red color represents the up-regulated genes, blue color represents the downregulated genes, gray color represents the non-DEGs (Normal cervix, n = 4; cervical cancer, n = 6). C) IHC scores for NOD1 and NOD2 in the early and advanced stage tumors (for NOD1, I-II stages: n = 39, III-IV stages: n = 53; for NOD2, I-II stages: n = 34, III-IV stages: n = 52). D) IHC scores of NOD1 and NOD2 in tumors of different grades (for NOD1, high and middle: n = 46, poor: n = 60; for NOD2, high and middle: n = 43, poor: n = 50). E) The positive correlation mRNA expression of NOD1 and NOD2 was identified by database (n = 306, http://timer.cistrome.org). F) Kaplan–Meier curves showing overall survival of CSCC patients demarcated on the basis of in situ NOD1 and NOD2 expression (http://www.proteinatlas.org). All data are presented as mean ± SD. *, P < 0.05; **, P < 0.01; ***, P < 0.001. [file 12916_2022_2248_MOESM1_ESM.tif]

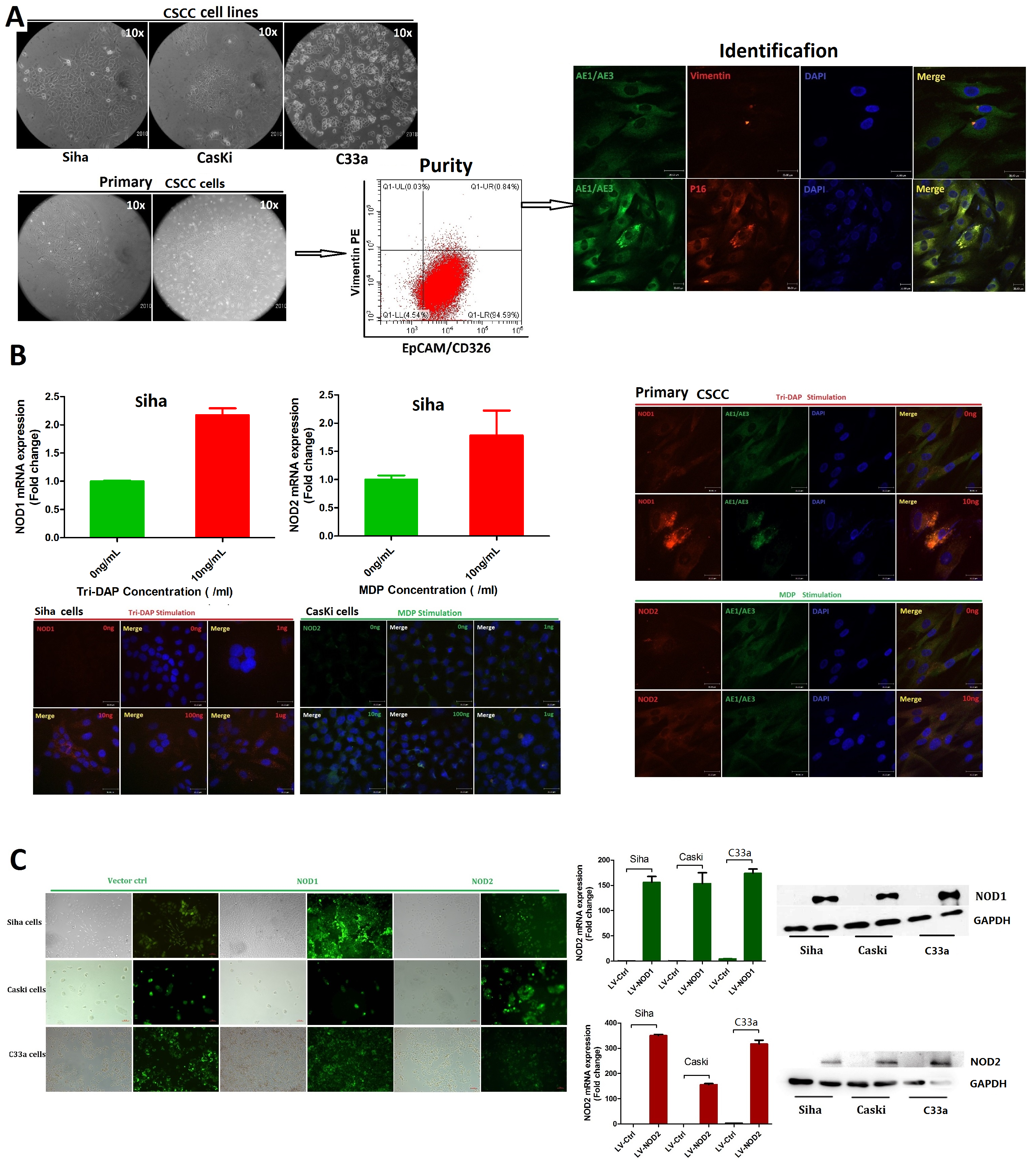

Supplement: Supplementary file 2 — Additional file 2. NOD1/2 expression in primary CSCC cells and CSCC cell lines. A) Morphology of the cultured Siha, Caski, C33a cell lines and the primary CSCC cells (left). EpCAM positive cells were sorted by MACS and identified by FCM (middle). The continuum of cultured primary CSCC cells were confirmed using immunofluorescence (AE1/AE3+ and P16+ and vimentin) (right). B) The level of NOD1 and NOD2 in CSCC cell lines and primary cells was upregulated through pretreatment by specific ligands (10 ng/mL) at 24 h by qPCR (upper) and in-cell immunofluorescence staining (lower). C) Stable NOD1 and NOD2 expression in the cultured Siha, CasKi and C33a cell lines were confirmed by green fluorescence (GFP flag), qPCR and western blotting. [file 12916_2022_2248_MOESM2_ESM.tif]

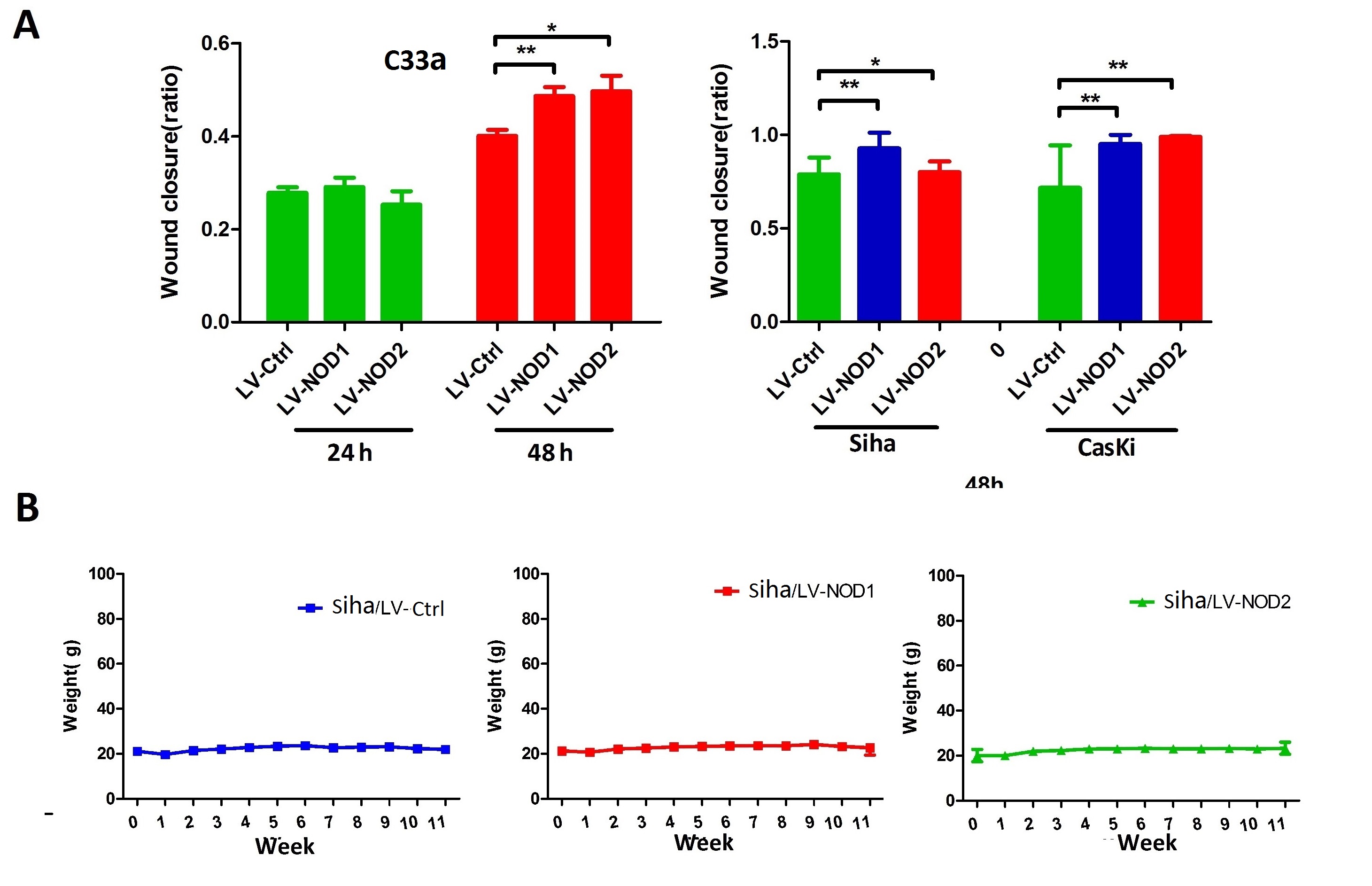

Supplement: Supplementary file 3 — Additional file 3. NOD1 and NOD2 enhanced the tumorigenic and metastatic abilities of CSCC cells. A) The percentages of wound healing, while data on C33a wound closure was normalized using the wound length at 0 h (left, 24 h and 48 h). The image on the right shows the percentage of wound closure of the Siha and CasKi cells, which were normalized to the wound length at 0 h (48 h). B) The weights of mice injected with different cell lines. ***, P < 0.001; **, P < 0.01; *, P < 0.05. [file 12916_2022_2248_MOESM3_ESM.jpg]

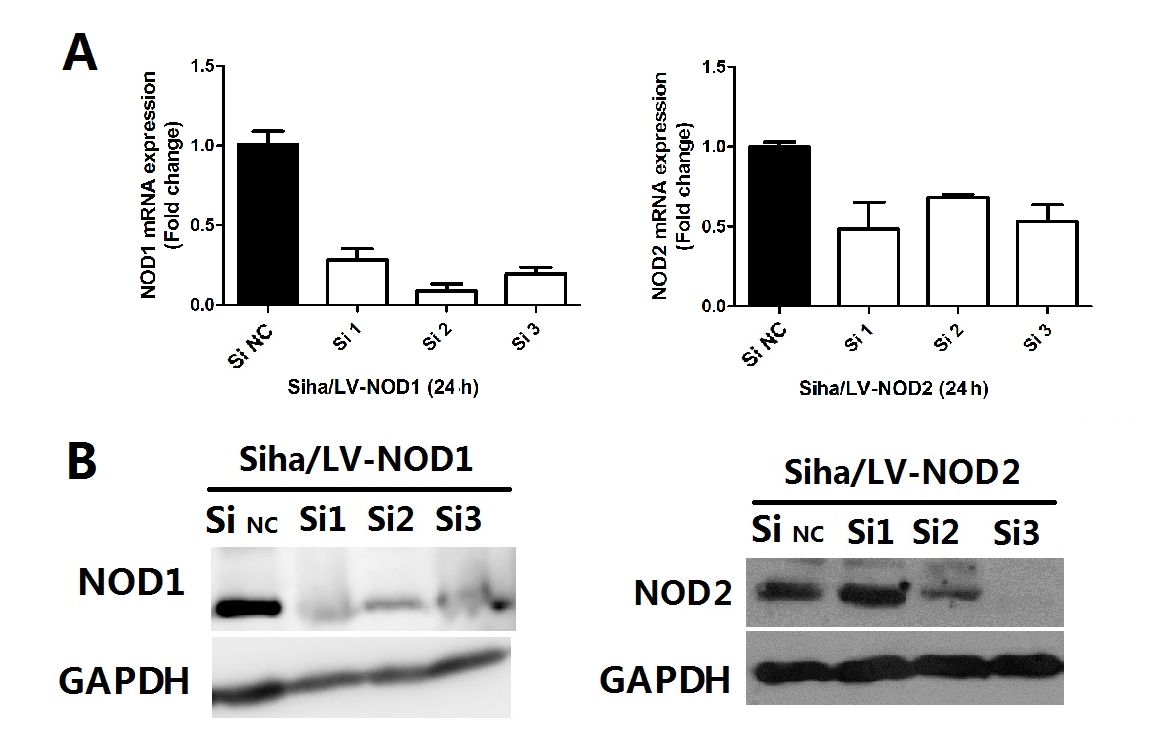

Supplement: Supplementary file 4 — Additional file 4. Siha/LV-NOD1 and Siha/LV-NOD2 cells transfected by siRNA. The expression of NOD1 mRNA (A, data were from three independent experiments with three replicates) and protein (B, the picture is a representative from two independent experiments). SiRNA was presented as Si1, Si2 and Si3. [file 12916_2022_2248_MOESM4_ESM.tif]

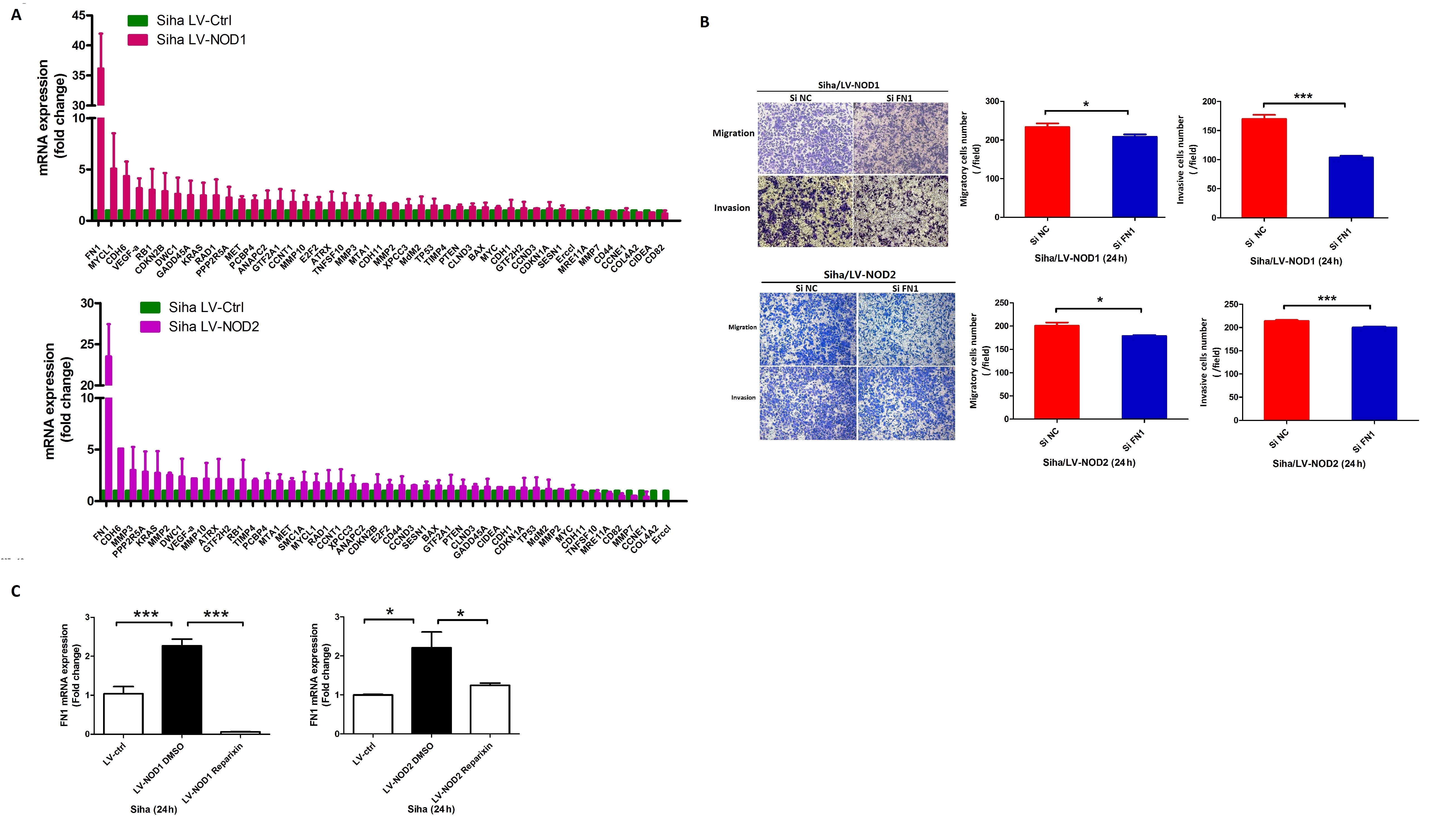

Supplement: Supplementary file 5 — Additional file 5. Over-expression of NOD1 or NOD2 of Siha cells promotes FN1 and IL-8. A) Representative results of adhesion and invasion molecules with over-expression of NOD1/NOD2. (Siha cells, two independent experiments using triplicated wells). B) Transwell assays revealed that the migration and invasion abilities of the Siha/LV-NOD1 and Siha/LV-NOD2 cells were inhibited by knock down FN1. C) Reparixin downregulates FN1 mRNA expression. [file 12916_2022_2248_MOESM5_ESM.jpg]
